# Design and Practical Evaluation of a Family of Lightweight Protocols for Heterogeneous Sensing through BLE Beacons in IoT Telemetry Applications

**DOI:** 10.3390/s18010057

**Published:** 2017-12-27

**Authors:** Dixys L. Hernández-Rojas, Tiago M. Fernández-Caramés, Paula Fraga-Lamas, Carlos J. Escudero

**Affiliations:** 1 Department Computer Science, Academic Unit of Civil Engineering, Universidad Técnica de Machala, Machala 070150, Ecuador; 2 Department Computer Engineering, Faculty of Computer Science, Universidade da Coruña, 15071 A Coruña, Spain; tiago.fernandez@udc.es (T.M.F.-C.); paula.fraga@udc.es (P.F.-L.); escudero@udc.es (C.J.E.)

**Keywords:** BLE, beacon, lightweight protocol, plug-and-play, smart sensors, IoT, WoT, telemetry, Eddystone

## Abstract

The Internet of Things (IoT) involves a wide variety of heterogeneous technologies and resource-constrained devices that interact with each other. Due to such constraints, IoT devices usually require lightweight protocols that optimize the use of resources and energy consumption. Among the different commercial IoT devices, Bluetooth and Bluetooth Low Energy (BLE)-based beacons, which broadcast periodically certain data packets to notify their presence, have experienced a remarkable growth, specially due to their application in indoor positioning systems. This article proposes a family of protocols named Lightweight Protocol for Sensors (LP4S) that provides fast responses and enables plug-and-play mechanisms that allow IoT telemetry systems to discover new nodes and to describe and auto-register the sensors and actuators connected to a beacon. Thus, three protocols are defined depending on the beacon hardware characteristics: LP4S-6 (for resource-constraint beacons), LP4S-X (for more powerful beacons) and LP4S-J (for beacons able to run complex firmware). In order to demonstrate the capabilities of the designed protocols, the most restrictive (LP4S-6) is tested after implementing it for a telemetry application in a beacon based on Eddystone (Google’s open beacon format). Thus, the beacon specification is extended in order to increase its ability to manage unlimited sensors in a telemetry system without interfering in its normal operation with Eddystone frames. The performed experiments show the feasibility of the proposed solution and its superiority, in terms of latency and energy consumption, with respect to approaches based on Generic Attribute Profile (GATT) when multiple users connect to a mote or in scenarios where latency is not a restriction, but where low-energy consumption is essential.

## 1. Introduction

Telemetry has been one of the driving forces behind the development of different technologies aimed at improving the efficiency of traditional automation applications. For instance, the Internet of Things (IoT) also depends upon the existence of reliable telemetry services. Moreover, the importance of IoT has risen remarkably in the last decade, with up to 15.4 bn IoT devices installed in 2015 that are expected to reach 30.7 bn in 2020 and 75.4 bn in 2025 [1].

Most of the current IoT systems are based on smart sensor networks that are mainly connected wirelessly, conforming what is known as a Wireless Sensor Network (WSN) [2]. Certain nodes of such networks are usually called motes due to their small size and limited computational power. Motes can be connected directly to the Internet or through gateways or border routers, being able to integrate web servers that allow for creating a web of sensors or Web of Things (WoT) [3].

After considering the available wireless technologies, this article focuses on Bluetooth Low Energy (BLE), which has become the ultimate choice of many IoT developers for the following main reasons:

- It has been embedded massively in smartphones.
- It is supported by the main operating systems (i.e., iOS, Android, Linux, OSX and Windows).
- It is a standard technology that is able to run IPv6 [4].
- Its features have been improved progressively. For instance, the latest specification, Bluetooth 5 [5], expands the coverage range from 50 to 200 m and its speed has been doubled with respect to Bluetooth 4.2.

In recent years, beacons, along with wearables, are the fastest pace-growing devices within the BLE ecosystem. BLE-based beacons are devices that broadcast specific data packets periodically in order to indicate its presence or to transmit certain information. They are commonly used in indoor navigation or in positioning systems. Some services [6] have reported that in the last quarter of 2016, 8 out of the 13 million sensors registered in their network, were beacons [7]. ABI Research latest forecast [8] predicts annual Bluetooth device shipments to reach 5 billion by 2021. Though smartphones will still account for 43% of Bluetooth device shipments at this time, Bluetooth Smart, also known as BLE, is exhibiting the strongest growth with a predicted 34% CAGR (Compound Annual Growth Rate) between 2016 and 2021, driven by new opportunities in beacons, home automation, and wearable applications in which lower energy consumption is critical. As a result, Bluetooth Smart Devices will account for 27% of total Bluetooth shipments by 2021.

Although beacons have many advantages (i.e., small size, light weight, low cost and low energy consumption), they are restricted in terms of memory, battery and hardware resources. Due to these reasons, beacons usually require the use of lightweight protocols in the different layers of the IoT stack that allow them to extend their battery life while decreasing latency and guaranteeing Quality of Service (QoS). These protocols have to minimize communications, computational load and storage needs in order to be implemented into beacons. Moreover, the use of standard protocols provides scalability, interoperability and functionality to current IoT systems. Therefore, such lightweight protocols have to be:

- Compatible with already existing protocols.
- Flexible to be implemented into beacons, wearables or more robust gateways.
- Scalable to support its application in new emerging fields.

Furthermore, the current needs of the IoT and WoT require these protocols to be able to use plug-and-play mechanisms to discover, describe and self-register the sensors and actuators connected to smart IoT devices. In addition, it is important to note that beacon technologies are small, unobtrusive and inexpensive hardware devices that enable data transmission and notify their presence to nearby Bluetooth devices. Therefore, they are able to transmit relevant, targeted messages and information to mobile devices within a specific range. They were conceived for applications different from telemetry, so they have restrictions with respect to the amount of sensors and actuators that can be managed without altering the way they operate.

In order to tackle the issues previously mentioned, this article proposes a solution that includes the following main contributions:

- It is defined a set of lightweight protocols (named LP4S, Lightweight Protocol for Sensors) that enable hardware auto-detection and auto-registration in the cloud. Furthermore, the proposed plug-and-play mechanisms are designed to be implemented in resource-constrained IoT devices.
- It is presented and evaluated a telemetry smart sensor based on a beacon based on Eddystone [9], an open BLE beacon format released by Google in July 2015. The solution makes use of the most restrictive LP4S protocol, which allows for creating telemetry applications with a large number of sensors and actuators without affecting the regular operation of the Eddystone protocol.

The rest of this article is organized as follows. Section 2 reviews the work related to lightweight protocols and BLE beacons. Section 3 describes the main elements of the LP4S family of protocols. Section 4 details the implementation of the LP4S-6 protocol in an Eddystone beacon. Section 5 describes and analyzes the results of several experiments that evaluate the performance of the LP4S-6 protocol. Finally, Section 6 is devoted to conclusions.

## 2. Related Work

### 2.1. Basic Requirements for IoT Devices

IoT has already enabled more effective monitoring, enhanced decision-making and effectiveness in sectors such as eHealth [10,11], smart cities [12], defense [13,14] and high-security applications [15,16,17], precision agriculture [18], transportation [19,20,21] or Industry 4.0 [22,23,24]. Current IoT systems are characterized by the use of resource-constrained electronic devices with reduced memory, limited communication capabilities, low computing power, and a high dependence on batteries. Therefore, they cannot support the implementation of complex security schemes [25]. These restrictions also apply to beacons and wearables, whose design represents a challenge for the IoT developer community [26,27]. In fact, some authors [28] describe ideal IoT devices as self-sufficient and energy-efficient computing elements that provide uninterrupted performance, QoS and long battery life. To create such ideal devices, the solutions devised have to focus on three main areas: energy efficiency [29,30,31], scheduling optimization and lightweight protocols. Thus, new lightweight protocols must be designed to reduce communications cost (i.e., to minimize the number of exchanged messages), computational cost (i.e., performing lightweight operations) and storage cost [32].

The work presented in this paper is focused on the development of new lightweight protocols that fulfill the requirements aforementioned and that implement plug-and-play mechanisms, as it will be further detailed in the next sections.

### 2.2. Lightweight Protocols for IoT Devices

The first lightweight protocols were aimed at reducing overhead in data transmission in the lower layers of the communications stack. However, as it is proposed in [33], it is not enough to make use of them at the physical and Medium Access Control (MAC) layers: lightweight protocols are also needed in the application layer, which allows for reducing power consumption while transmitting the necessary messages. Thus, in recent years, a lot of progress has been made and lightweight protocols can be found for practically every layer of the communications stack.

Regarding the MAC layer, Chen et al. [34] present an interesting proposal for developing a lightweight WSN MAC protocol for a smart home. Such a protocol replaces IEEE 802.15.4, which is complex and has a low packet-delivery ratio. The authors tested the protocol by using an Field-Programmable Gate Array (FPGA) and were able to increase packet-delivery ratio up to 100%. Other researchers [35] contributed to several layers of the stack with implementations for Contiki [36] when making use of Constrained Application Protocol (CoAP) [37] in 6LoWPAN (IPv6 over Low power Wireless Personal Area Networks) [38] connected Bluetooth Smart devices.

With respect to the network layer, one of the first revisions of the lightweight protocols for IoT is presented in [39]. In the paper, the authors emphasize the role of 6LoWPAN and carry out an implementation that includes the use of Representational State Transfer (REST) services and Tiny OS-based nodes. Another solution is presented in [40], where network and security layer protocols are combined, integrating Datagram Transport Layer Security (DTLS) [41] with 6LoWPAN in the so-called 6LoWPAN-NHC, which encodes different DTLS headers. The researchers implemented the CoAP-based system in Contiki, although they state that it can be implemented in any other operating system that supports 6LoWPAN.

The development of secure lightweight authentication techniques is still a challenge for the IoT community, as it shall achieve a balance between the high security level, efficiency, computational and communication cost. Some authors have already addressed some of such challenges [42]. For instance, in [43] it is presented a robust CoAP-based authentication scheme for monitoring resources in an IoT environment. Another interesting example can be found in Reference [44], where it is proposed the use of a lightweight cryptography protocol based on XOR Operations. Additionally, it is worth mentioning that some of the latest reviews on lightweight authentication protocols for wearables can be found in Reference [32,45,46].

Among all the layers, the application layer is probably the one that has had recently more impact on the rise of the IoT thanks to the development of several successful lightweight protocols such as Message Queue Telemetry Transport (MQTT), CoAP, Extensible Messaging and Presence Protocol (XMPP) and Advanced Message Queuing Protocol (AMQP) [28,33,47,48,49].

Several authors have proposed protocols for the application and the presentation layers. For example, in order to obtain a lightweight protocol for the WoT, Cheah et al. [50] implement the Web Thing Model (WTM) proposed by the W3C [51] in CoAP-based prototypes that discover and describe the different elements of a smart home environment. Another interesting proposal is the Lightweight Machine-to-Machine (LWM2M) protocol, which is supported by the Open Mobile Alliance (OMA) and is described in [52] (an example of implementation can be found in [53]).

The contribution of the aforementioned works in the different layers of an IoT stack is summarized in Table 1. Although many of these works have cross-layer approaches, they have been grouped into specific layers. For a better comprehension, some of the protocols prevailing in each layer are included, even though the protocol presented in this article is not focused on the analysis of such layers, but in the application and presentation layers.

### 2.3. Bluetooth Low Energy Communication Basics

In the last few years, different technologies have been proposed to provide wireless connectivity to WSNs and to enable IoT sensor-based telemetry frameworks such as Bluetooth [54], Zigbee [55], Wi-Fi, Low Frequency (LF)/High Frequency (HF)/Ultra-High Frequency (UHF) RFID [56,57,58], or even multi-technology solutions [59].

Among them, Bluetooth is one of the wireless technologies with the highest growth and penetration in the electronics, telecommunications and consumer industries. Its presence in peripherals such as keyboards, mouse, hearing aids and smartphones have guaranteed its use in the short and medium term. Three Bluetooth versions can be distinguished: BLE, Bluetooth Basic Rate/Enhanced Data Rate (BR/EDR) and Dual Mode (BR/EDR/BLE). A device that implements the latter can communicate both with BR/EDR and BLE devices. BR/EDR is known as *Classic Bluetooth*, while Dual Mode is called *Smart Ready* and BLE is named simply *Smart*. Each implementation has been devised for specific uses and different chipsets [60].

The different implementations follow the specifications issued by the Bluetooth Special Interest Group (SIG), which has released versions 2.0 and 2.1 for BR/EDR and 4.0, 4.1, 4.2 and 5.0 for BLE.

It can be stated that BLE is the IoT version of Bluetooth, since its low energy consumption is perfect for battery dependent devices, whether they are powered by coin-cell/AA/AAA batteries or by some kind of energy-harvesting solution.

BLE systems work at the unlicensed 2.4 GHz ISM band and use frequency hopping to minimize the interference from other RF devices that operate at the same band (e.g., Wi-Fi or classic Bluetooth). In order to communicate BLE devices with each other, one of them has to take the role of advertiser and the other one, the role of scanner. The advertiser sends advertising packets so that a scanner can discover them and establish a communication between both. Once the communication is established between the devices, they must adopt the roles of master and slave during the data exchange. Advertisement packets are sent at specific time intervals through the channels 37, 38 and 39 of the 2.4 GHz band. The v5.0 specification [5] defines these periodic packets as events that happen after a certain number of time units. Four types of events are defined: Advertising, Extended Advertising, Periodic Advertising and Connection events.

There are two basic kinds of communications of BLE devices in IoT: the first one is performed through the Generic Attribute Profile (GATT) with its services and characteristics, while the second one is related to beacons. The first communication method requires establishing a connection with the BLE device discovered, while beacons are connectionless (i.e., they just need to be discovered). The protocols proposed in this article are designed to work both on GATT and beacon communications, as it will be explained later in Section 3. A deep description of the BLE standard is beyond the scope of this paper, but the interested readers have an excellent starting point in [60].

### 2.4. BLE Beacons

**Figure 1:**
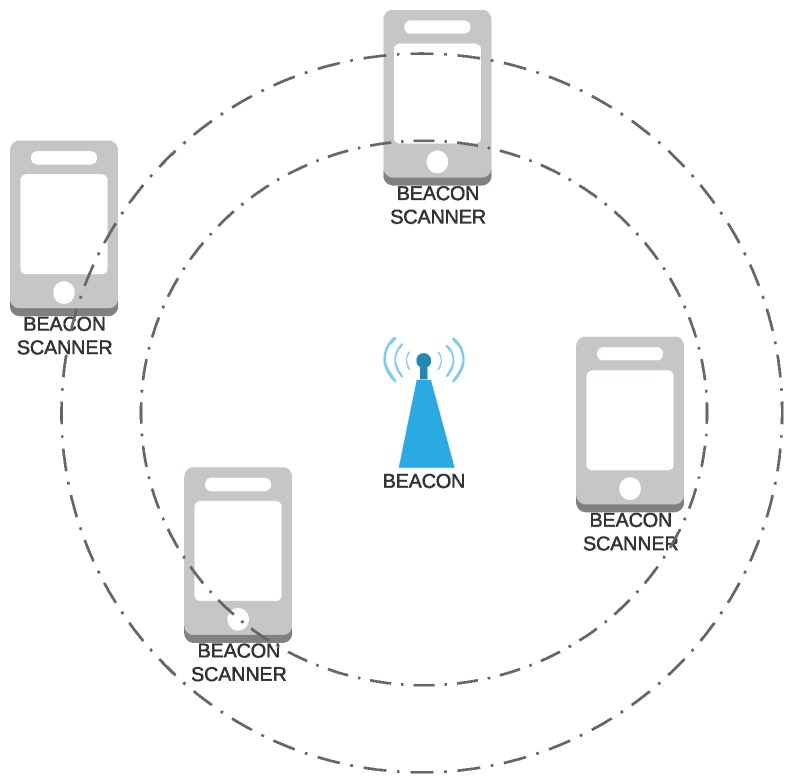
Scenario with multiple beacon scanners.

BLE beacons are small devices powered by batteries that are able to broadcast certain information (e.g., position, url, battery status, the values of attached sensors) without establishing a connection with a BLE scanner. This information is sent in the advertising packets according to the SIG specifications. For instance, a smartphone acting as a scanner may be able to obtain from the beacon, its location, and it can generate notifications (i.e., alerts), receive messages or activate services when approaching it [61]. A typical beaconing scenario is illustrated in Figure 1, where broadcast messages from a beacon are read by multiple scanners (i.e., receivers).

**Figure 2:**
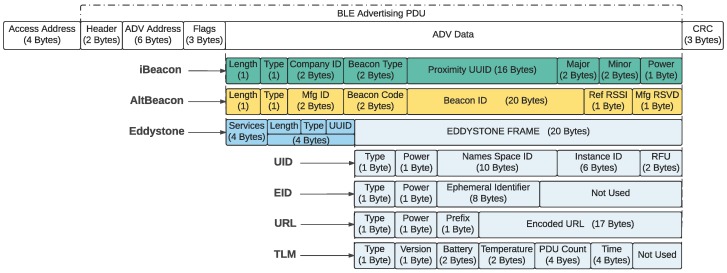
Structure of iBeacon, AltBeacon and Eddystone beacon protocols.

Although Bluetooth SIG has not defined an official beaconing standard, there are three main implementations: iBeacon [62], Altbeacon [63] and Eddystone, which have been defined by Apple, Radius Network, and Google, respectively. Figure 2 shows the internal structure of the packets sent by such protocols.

The iBeacon family were the first beacons to enter the market. They were designed under the closed-source philosophy of Apple, conceived to be used only with iPhones, iPads and iPods through its iBeacon certification program. Nevertheless, many people began to use iBeacon technology on Android devices despite these restrictions. This trend led Radius Network to create an open-source beacon called Altbeacon, which differs from iBeacons in their packet structure, but that is compatible with them and provides similar functionality. Two years after launching the first beacons, Google entered in the market with its Eddystone beacon, backed by its broad Android community. Google, unlike Apple, provides support for Eddystone for the iOS operating system. It is important to note that, as it is shown in Figure 2, the Eddystone protocol makes use of four frames:

- UID (Unique-ID) frames: they broadcast a unique code that, when associated with certain information in an application, it can be useful to identify locations or objects.
- EID (Ephemeral ID) frames: they are a variant of the UID frames that obfuscate the ID for greater privacy and security. This frame has not been used widely except by some manufacturers such as Gimbal [64], Swirl [65] and Shopkick [66], consequently it will not be considered in the rest of the manuscript.
- URL frames: they broadcast a URL that points to a Secure Sockets Layer (SSL) protected website.
- TLM (Telemetry) frames: they broadcast information about a beacon, like its battery level or the values of the embedded sensors. Since this paper is focused on telemetry applications, these are the most important frames.

Regarding beacon manufacturers for the IoT market, there is a large number of them, which are competing for positioning their products in different applications in terms of lower prices, smaller sizes and longer battery lifes. Table 2 compares the features of some of the most popular commercial beacons (more devices and an excellent overview of the history of the beacon technology is presented in [67]). In such a Table it can be observed that the majority of the chipsets are manufactured by two technology giants, Nordic Semiconductor and Texas Instruments (TI) and, to a lesser extent, by Bluegiga and Qualcomm (that acquired CSR). Regarding the protocols supported, Eddystone and iBeacon prevail over the other solutions.

Extensive lists of commercial beacons can be found in [78,79]. In such lists Kontak.io stands out as the leader in the hardware category, followed by Estimote, while Swirl leads the proximity platform category [7].

### 2.5. Beacon Applications

Beacons have been designed for opportunistic data collection [4], mainly to estimate reading distance and proximity, and to transmit notifications. Thanks to such capabilities beacons have been useful in retail, entertainment, museums, or even airports [80]. Moreover, research has been focused on analyzing navigation and tracking techniques [81,82], indoor positioning schemes [47,83,84] and proximity notifications [85,86]. Other applications are the implementation of the physical web to obtain positions [87] or to enable educational web pages [88].

An extensive review of 100 beacon-based use cases is presented in Reference [89]. Most of the applications are focused on proximity marketing solutions. This fact indicates that beacons have not being fully exploited in terms of added value in other applications, like in the case of telemetry applications. One of the reasons is that, although the Eddystone protocol can broadcast telemetry frames, they only contain two specific telemetry fields (e.g., the battery status and a temperature value), what limits the number of sensors to be notified. For instance, one of the few examples of beacon-based telemetry is presented in Reference [90], where the status of a on/off digital input is broadcast.

There are preliminary works that encapsulate new protocols into a beacon standard protocol. For example, in Reference [91] it is proposed an interleaving technique that makes use of multiple BLE advertisements that identify devices based on the Altbeacon protocol. Another interesting patent is Reference [92], where it is proposed a technique that allows for identifying devices connected to iBeacons. Such an identification is performed by transmitting successive patterns at different power levels. Then, it is possible to identify and calibrate the connected device through its power pattern.

The lightweight protocol presented in this paper (Lightweight Protocol for Eddystone, LP4Sensors) can be encapsulated into the standard frames of an Eddystone Beacon. The protocol allows for the transmission of telemetry data from unlimited heterogeneous analog and digital sensors, limited only by the pinout of the beacon hardware. The protocol is inserted into Eddystone beacon frames without affecting the standard and requiring only 6 bytes. Furthermore, it is able to identify the sensors and actuators connected, even including an information about them or their pinout. In addition, the discovery and register of transducers in a IoT system may be carried out through a plug-and-play mechanism.

## 3. Lightweight Protocol for Sensors (LP4S)

In this Section it is presented LP4S, a lightweight family of protocols that can:

- Be embedded into BLE beacons.
- Be used to send the data collected from the beacon sensors and to receive commands for the actuators.
- Guarantee the QoS required.
- Provide very low power consumption.
- Be able to discover and describe the hardware embedded automatically by using plug-and-play techniques.

Although the LP4S protocol was designed to be used primarily for telemetry, monitoring, control and positioning systems, its flexibility allows it to be extended to other applications that require to communicate resource-constrained devices.

The LP4S protocol defines three types of frames:

- Configuration frames. They are used to describe the hardware of the beacon, allowing it to auto-register an IoT systems through plug-and-play mechanisms.
- Data frames. They are used to send the actual values of the sensors and the status of the actuators, whether they are digital or analog. They are also used to send unidirectional commands to the motes.
- Bidirectional command frames. This kind of frames is optional and depends on the resources and the type of beacon used. For example, this type of frame makes no sense in certain beacons that only work in off-line mode, where they only send information on each advertisement. However, when a bidirectional communication is required, these frames can be used to send commands to the beacon, which will send a confirmation of the received command towards the server, gateway or device that generated the command.

The protocol has to fulfill the following three requirements in order to be considered lightweight:

- Low storage cost or low footprint. The developed firmware cannot occupy much memory and it should be possible to upload it to resource-constrained beacons.
- Low computational cost. Frames have to be short, which allows for speeding up computational tasks at both ends of the communications link.
- Low communication cost. The number of frames to be transmitted has to be minimized as much as possible to reduce communications cost in terms of energy consumption.

Given the diversity of IoT beacons currently available, which differ in terms of hardware constraints and communication technologies, it was necessary to create a family of protocols that allows for fulfilling the requirements detailed previously. For such a purpose, three groups of beacons were defined according to their resources:

- Type-1 beacons. They are very limited in terms of hardware resources and protocol complexity (i.e., the amount of bytes and frames to be processed is really restricted). This is is the case of most beacons.
- Type-2 beacons. They have more hardware resources than Type-1 beacons and are able to handle larger frames. This is the kind of beacons that offer GATT data through BLE communications.
- Type-3 beacons. These are IoT devices that, although powered by batteries, they have more computing and memory resources that allow for running more complex firmware and for processing web-type data, such as JavaScript Object Notation (JSON) files.

Thus, the LP4S family is composed by three sub-protocols:

- LP4S-6: it is used for Type-1 beacons.
- LP4S-X: it is used for Type-2 beacons.
- LP4S-J: it is used for Type-3 beacons.

As it will be explained in the next sections, each of these three protocols makes use of a set of frames that has been designed looking for a balance between the amount of bytes to be transmitted, the number of frames involved in the communications and the complexity of such frames.

### 3.1. LP4S-Six (LP4S-6)

This protocol only makes use of configuration and data frames, since it was designed from its inception to be used in Eddystone beacons. However, the protocol is not limited to such a kind of beacons, being able to be encapsulated into other existing standard beacons or proprietary protocols, and even in serial communications between chips. Note that the main restriction of Type-1 beacons is the size of the protocol frames, so it was necessary to design a protocol that met the desired requirements in only 6 bytes.

**Figure 3:**
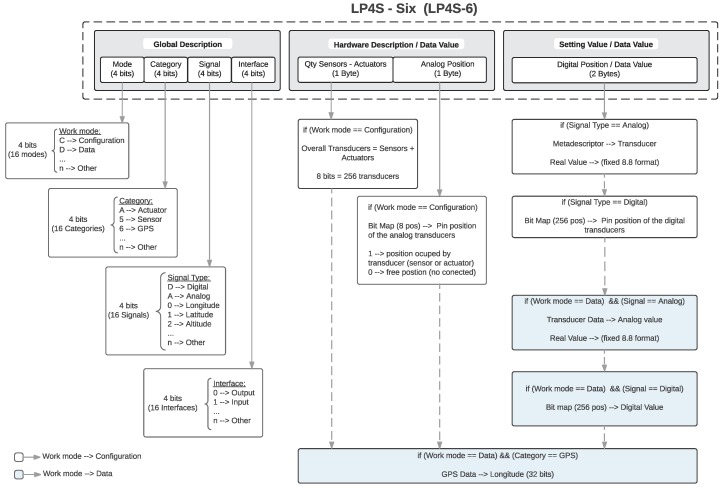
Internal structure of the LP4S-6 protocol.

Figure 3 shows the structure of this protocol. It can be observed that the protocol is formed by three groups of two bytes. Each group has more than one functionality depending on the type of the information managed (i.e., a description, a configuration or or certain data). The first two-byte group (Global Description), contains general details about the beacon and the information that is sent. Such an information is divided into four nibbles that include the work mode, the type of embedded transducer, the kind of signal transmitted and the type of interface. Thus, it is possible to indicate if the information sent is related to the configuration or the data of a specific sensor, whether it is digital or analog, or if the data transmitted are related to inputs or outputs. Since each node enables the use of up to 16 different descriptions (some of which have not been defined yet), it can be stated that the protocol is flexible, scalable and open to future improvements and customization for new IoT applications.

The second two-byte group (Hardware Description/Data Value), has a content that depends on the information included in the previous group:

- When the work mode on the Global Description indicates that a configuration frame is being sent, then the first byte contains the total number of active sensors and actuators in the beacon. Among other functionality, this activity field allows remote users to indicate whether they have received all the information from the beacon. Moreover, in this work mode, the second byte is a 16-bit map indicating (when is equal to “1”) when a transducer is connected and operating.
- In data mode both bytes contain the value read from the transducer specified in the Global Description.

The third two-byte group (Setting Value/Data Value) also depends on the content of the Global Description:

- In the case of configuration frames, this field indicates the position and the data type of transducer (i.e., analog or digital).
- When a data frame is transmitted, the sensor value is indicated with a precision of 16 bits. If the embedded transducer is analog, then the two bytes contain a real value in a fixed-point 8.8 format. In the case of digital sensors, the information is provided in the form of a 16-bit map. If what is being reported is a GPS position, then a “1” indicates that the transducer is active. Finally, if what is reported is a digital value, then a bit map will be given where the bit related to the position of the digital sensor will be set to “0” or “1” depending on its state.

**Figure 4:**
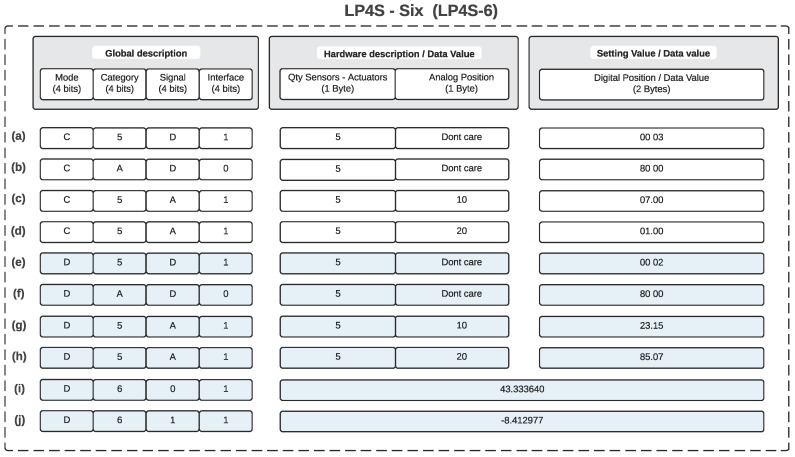
Examples of use of the LP4S-6 protocol. (**a**–**d**) Configuration frames: digital and analog sensor and actuators, (**e**–**h**) Frames with sensor data, and digital and analog actuators, (**i**–**j**) Localization frames: longitude and latitude.

For a better understanding of the protocol, several examples are shown in Figure 4. Case (a) represents a configuration frame that indicates that there are a total of 5 transducers, two of which correspond to digital sensors located at positions 0 and 1 of the map (0x0003 in hexadecimal is equal to 0000 0000 0000 0011 in binary). In the example (b) it is indicated that there is a digital output located at the last bitmap position (0x8000, 1000 0000 0000 0000). Examples (c) and (d) represent configuration frames where two sensors connected to analog inputs located in the fifth (0001 0000) and sixth position (0010 0000) are used. Moreover, in these two last examples, the associated meta-descriptors (7.00 and 1.00) represent indexes of a table that contains a more detailed description of the transducers. For example, the sensor identified by the meta-descriptor 7.00 might represent a temperature sensor given in °C, while the one identified by 1.00 might be a relative humidity sensor whose measurement unit is a percentage.

Examples (e) to (j) show use cases where it is sent the information collected by the sensors previously described in (a) to (d). In (e) it is indicated that, of the two digital sensors defined in (a), the first digital input is set to “0” and the second one, a logical “1” (for instance, due to the activation of a PIR sensor or when opening a door). The example (f) indicates the status of a digital output that is set to “1”. Use cases (g) and (h) broadcast real-time values of two analog inputs, which, according to the configuration frames previously sent in (c) and (d), and the assumptions indicated about their sensor type on a table, they would indicate a temperature of 23.15 °C and a relative humidity of 85.07 %. Finally, (i) and (j) show the current geographical position of the beacon, which is would be located at coordinates (43.33640, −8.412977).

#### 3.1.1. LP4S-Extended (LP4S-X)

**Figure 5:**
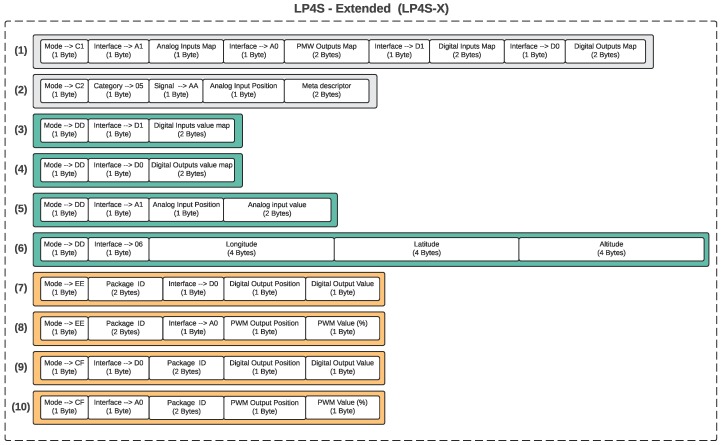
Internal structure of the LP4S–Extended (LP4S–X) protocol (1–2) Configuration frames, (3–5) Reading frames for digital, analog and location data, (7–8) Frames for digital and analog commands, (9–10) Acknowledgment frames.

This protocol is a variation of the LP4S-6 protocol that has been modified to avoid the restriction of having only 6 bytes per frame. Therefore, it constitutes an extension of the previous protocol (hence its name). Although there are no restrictions on the number of bytes, only two of the ten LP4S-X frames have actually more than 6 bytes (some of them contain only 4 bytes). This protocol makes use of the three basic types of frames depicted in different colors in Figure 5, which are related to configurations, data exchanges and bidirectional commands.

Each of the frames starts with one byte that allows for identifying the frame type: C1 and C2 represent configuration frames, DD are data frames, EE are command frames and CF, confirmation frames.

As it can be observed, there are two configuration frames in this protocol, C1 and C2. The former type, whose structure in depicted in Frame (1) in Figure 5, is used for describing the position of all input and output transducers (both analog and digital) through bit maps, similarly to LP4S-6. In Figure 5, in Frame (2), it is shown the structure of C2 configuration frames, which are used for describing each transducer. Therefore, there will be sent as many C2 frames as transducers with descriptive requirements are connected to the beacon. Although C2 frames are typically used for analog sensors, they can be used to describe any other transducer that requires a detailed description.

Frames (3) and (4) of Figure 5 inform about the status of the digital inputs and outputs by using bitmaps. In the case of analog sensors, it is also necessary to send a frame like (5) in order to indicate the sensed values. Regarding Frame (6), it embeds into a single frame all the geolocation information (longitude, latitude and altitude) about a position.

Frames (7) and (8) are used to send commands to beacon actuators, whose response can be controlled (e.g., relays, LEDs or Pulse-Width Modulation (PWM) controllers). Since such frames do not use bitmaps, a frame must be sent for every digital or PWM output to be modified.

Finally, Frames (9) and (10) represent frames that are sent by the beacon to the server in order to confirm that a command was received. Such frames include an ID field, unique for each message, which helps the server to identify the command acknowledged. These frames are also used to send the current status of an output modified previously, what allows for establishing alarms or retransmission mechanisms at the server side.

### 3.2. LP4S-JSON

The third and final protocol of the LP4S family has been designed for beacons that have access to a relevant amount of resources. In such devices it is possible to implement protocols like CoAP, which usually exchange information in JSON format. In LP4S-JSON there are three frame types:

- LP4S—Configuration (LP4S-JC): Configuration frame.
- LP4S—Read (LP4S-JR): Data frame and LP4S.
- LP4S—Write (LP4S-JW) Command/confirmation frame.

Each of these frames contains data in JSON format that make use of the tags shown in Table 3 in order to create a semantic representation of the beacon. Such a representation allows for describing their hardware and for discovering and self-registering transducers through plug-and-play mechanisms. Some of the JSON tags in Table 3 indicate the name of the beacon, its MAC address, different meta descriptors or the bitmap positions of the digital sensors. There is even a tag to notify a BLE scanner when a button is pressed.

#### 3.2.1. LP4S-JSON Configuration (LP4S-JC)

Like in the other protocols of the LP4S family, the configuration formats designed enable the description of the existing hardware and facilitate the easy discovery and registration of the hardware in IoT applications. Thanks to the tags in Table 3 it is possible to perform such discovery and registration tasks with a semantic description that can be adapted to the WoT.

**Figure 6:**
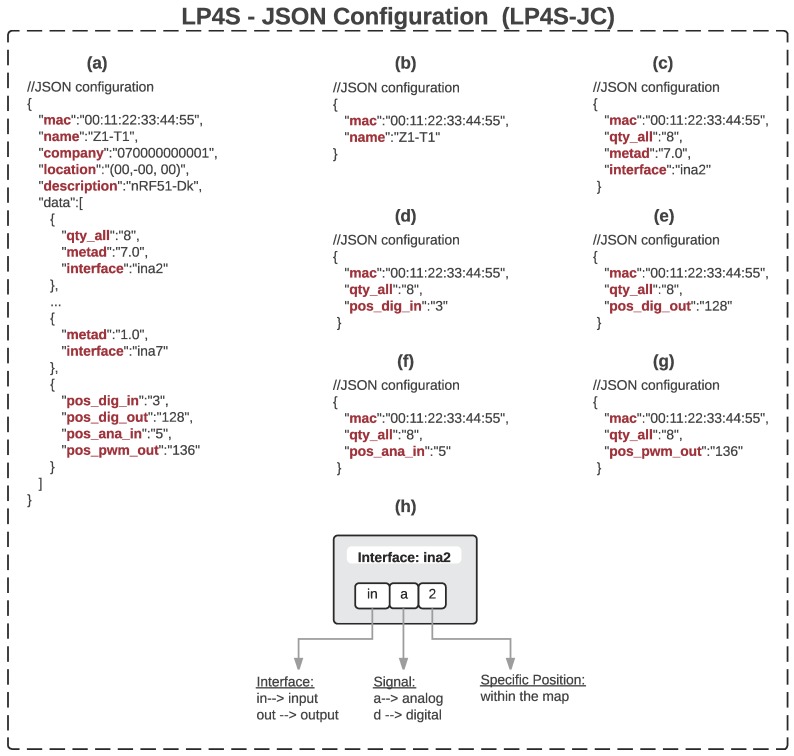
LP4S-JC messages that show JSONs that include all the tags (**a**), a subset of the tags (**b**–**g**), and a mixed type parameter (**h**).

Several examples of use of the LP4S-JC protocol are shown in Figure 6. Note that, in order to maintain the lightness of the protocol, it is possible to send a micro-JSON per configuration parameter (i.e., in Figure 6b–g) instead of sending all parameters (like in Figure 6a) in a single JSON. It is important to note that each micro-JSON always includes the MAC address of the beacon as a parameter, which guarantees the identification of the device. Finally, in (h) it is illustrated an example of a mixed type parameter that represents an input (‘in’) analog (‘a’) sensor located in the second position (‘2’ in decimal or “0000 0000 0000 0010” in binary) of a bitmap. This kind of types are used to reduce the number of JSON tags and maintain the lightness of the protocol in certain applications.

#### 3.2.2. LP4S-JSON Read (LP4S-JR)

This protocol is used to represent the data frames that the beacon sends when the value of some of its sensors has changed. However, the beacon firmware could be modified to use other criteria to trigger the packet broadcast.

**Figure 7:**
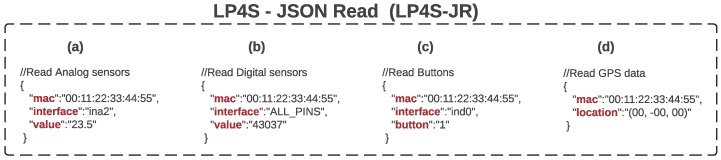
Examples of use of LP4S-JC: (**a**) analog data, (**b**–**c**) digital data and (**d**) geolocation data.

LP4S-JR makes use of micro-JSONs whose structure is shown in Figure 7. In such a figure, in (a) it is represented a case where it is broadcast a temperature of 23.5 °C of the sensor located in position 2. In the example in (b) it is shown the current status of all the digital inputs and outputs (note that the special tag “ALL PINS” was used). Regarding the example (c), it illustrates a notification to a server when the button in the first position was pressed. Finally, in (d) it is shown the JSON for sending the geolocation coordinates of the beacon.

#### 3.2.3. LP4S-JSON Write (LP4S-JW)

**Figure 8:**
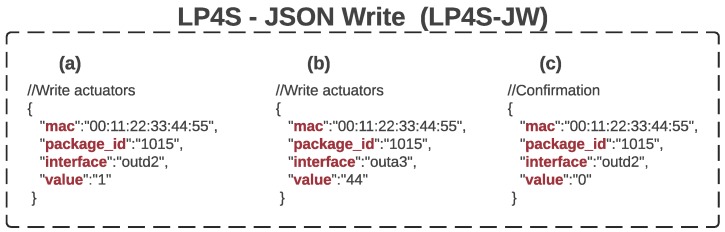
Examples of use of LP4S-JC. (**a**) digital command, (**b**) analog command, (**c**) confirmation message.

The structure of the command and confirmation messages is shown through examples in Figure 8. In the case of (a), it is represented a command that activates a digital actuator (for instance, a relay or an LED) that is connected to the second position (‘outd2’) of the beacon. The example (b) shows a command that changes the PWM output located in the third position (‘outa3’) to 44%. Finally, the example (c) contains the confirmation message that would be sent by a beacon after receiving the command shown in (a). Note that, in this last example (c), the value of “package_id” has to match the one in (a). However, note that in (c) the state of ’value’ is ‘0’, while in (a) it was indicated that it had to be set to ‘1’, what denotes that the command was not processed or it was processed erroneously. In such a case, the server would react according to the programmed logic (for instance, it might retransmit automatically the command).

## 4. Implementation of the LP4S Protocol on a Beacon

**Figure 9:**
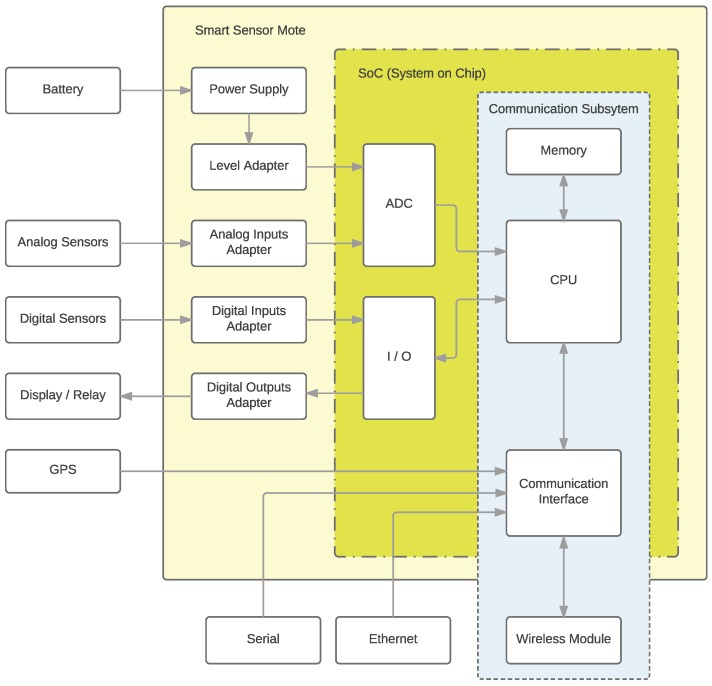
Generic smart sensor mote.

The LP4S family of protocols can be implemented in any beacon or mote whose hardware follows the architecture illustrated in Figure 9. Note that such an architecture is just a reference, so certain difference can exist in terms of hardware, but the protocol may still be compatible. For instance, the communications subsystem might be integrated into the main System-on-a-Chip (SoC) of the beacon or in an external module.

**Figure 10:**
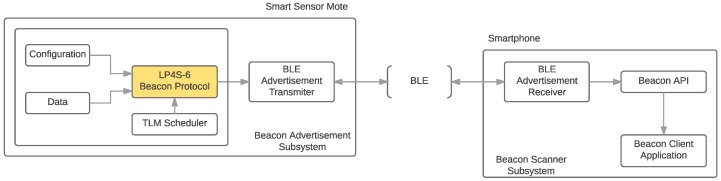
Communications architecture.

In this Section it is specifically addressed the implementation of the LP4S-6 protocol in a resource-constrained beacon like the Eddystone. Such a beacon transmits the certain information periodically with each BLE advertisement. Figure 10 shows the communications architecture of a generic beacon system and an illustration on how the LP4S-6 protocol is inserted into the scheduler of the TLM frames. Any application running on a beacon scanner (e.g., on a smartphone or on a Single-Board Computer (SBC)) that implements the API proposed in this paper is able to read both conventional Eddystone frames and the telemetry information encapsulated by the LP4S-6 protocol. Moreover, since a regular Eddystone scanner is not be affected by the modifications performed by the LP4S-6, it will continue to read a beacon that make use of both protocols. Therefore, a beacon that implements the LP4S-6 protocol can work in a beacon broadcasting network with the presence of multiple and different beacon scanners from different manufacturers and different applications without interference among them.

**Figure 11:**
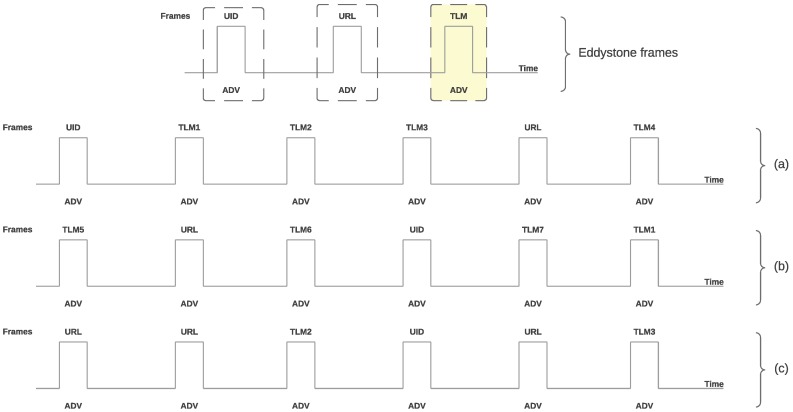
Examples of Eddystone frame sequences.

Before detailing the protocol implementation, it is important to note that the designed firmware broadcasts three out of the four Eddystone frames (UID, URL and TLM) in a random order and according to a TLM schedule, as depicted in Figure 11 to illustrate the transmission scheme. In these three examples of possible frame sequences, it can be observed that the frames are sent cyclically, (i.e., once the last frame was sent). The cycle is started by the first frame sent according to the schedule established, which depends on the number of embedded sensors and on the availability of sensed data.

### 4.1. LP4S-6 on a Eddystone Beacon

The LP4S-6 protocol was designed to be inserted into protocols restricted in size, such as Eddystone, and which also use of pre-established frame sizes and frames. Specifically, in each Eddystone TLM frame it is possible to find 6 bytes that are not currently used by the Eddystone specification, where the LP4S-6 protocol fits perfectly. In this way, it is possible to adapt traditional Eddystone beacons to be used in telemetry applications that do not require a connection for their operation, what is also useful in certain IoT applications.

**Figure 12:**
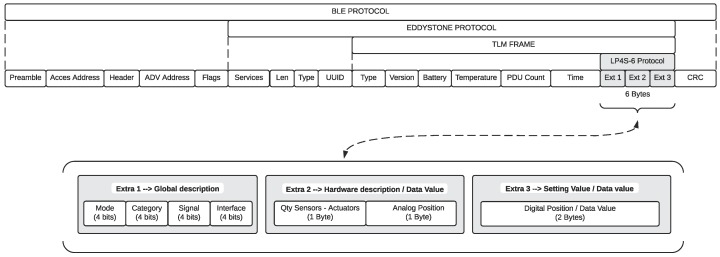
LP4S-6 inserted into a TLM frame.

In Figure 12 it is indicated the precise location of the Eddystone TLM frames where the LP4S-6 protocol is inserted. The space that hosts the protocol proposed was divided into three pairs of bytes (Extra 1, Extra 2 and Extra 3).

### 4.2. Workflow Diagrams

This Section describes the flow diagrams needed to implement telemetry applications in commercial beacons, both supporting the Eddystone protocol and the LP4S-6, which adds the value of being able to use unlimited sensors (obviously, such a limit depends on the amount of analog and digital input/output pins available on the selected hardware).

**Figure 13:**
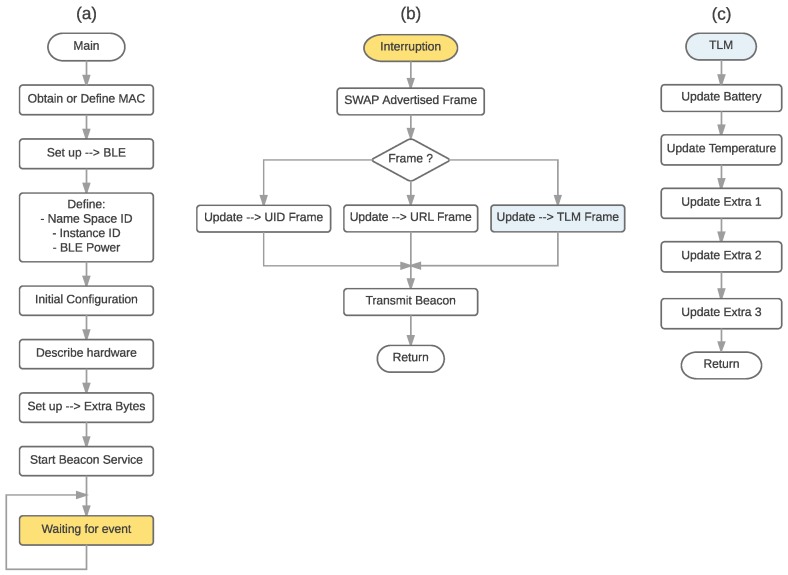
Beacon flow diagrams. (**a**) Main, (**b**) Advertisement Interrupt Service Routine (ISR), and (**c**) TLM flow.

Figure 13a shows the flow diagram of the main program of a conventional beacon, whose main tasks are the configuration of the hardware, and the initialization of the parameters and services of the beacon to carry out BLE communications. After the initialization, the beacon waits for the interruptions generated by a timer to begin to exchange frames following the flow illustrated in Figure 13b. When it is time to broadcast a TLM frame, the different fields of a standard Eddystone frame and the extra bytes of the LP4S-6 protocol are updated according to the flow shown in Figure 13c. In the flow diagram two specific parameters are indicated in every TLM frame (the battery level and the ambient temperature), which are updated periodically by a parallel thread and stored in a shared memory area.

**Figure 14:**
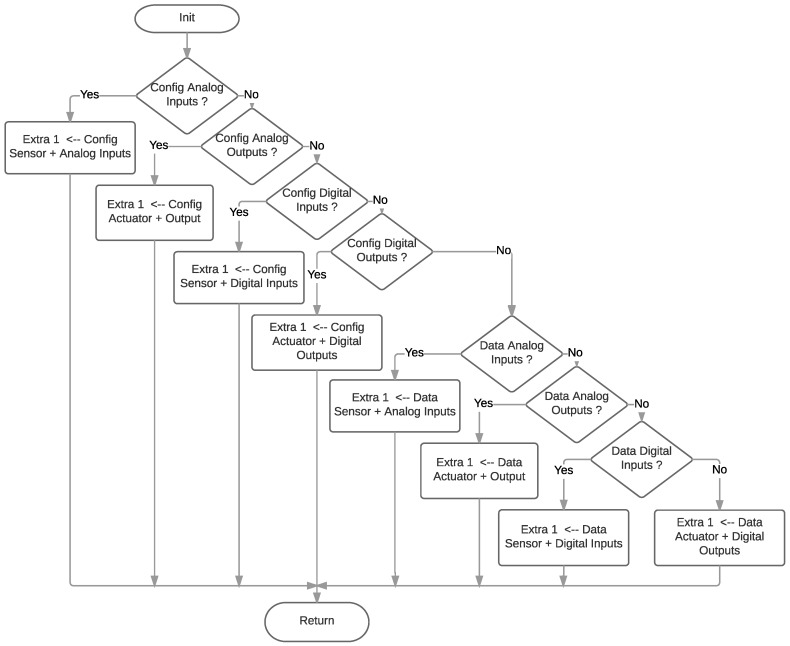
Extra 1 diagram flow.

As explained in Section 3.1, the fields of the LP4S-6 protocol are multi-functional, so it is necessary to implement an algorithm that controls the information required in each of the frame types to be transmitted. Figure 14 shows the flow diagram that illustrates how the Extra 1 byte is implemented. Such a byte corresponds to the Global Description field of the LP4S-6 protocol, so the algorithm allows for filling in the fields related to the Mode (Configuration or Data), Category (Sensor or Actuator), Signal (Analog or Digital) and Interface (Input or Output).

**Figure 15:**
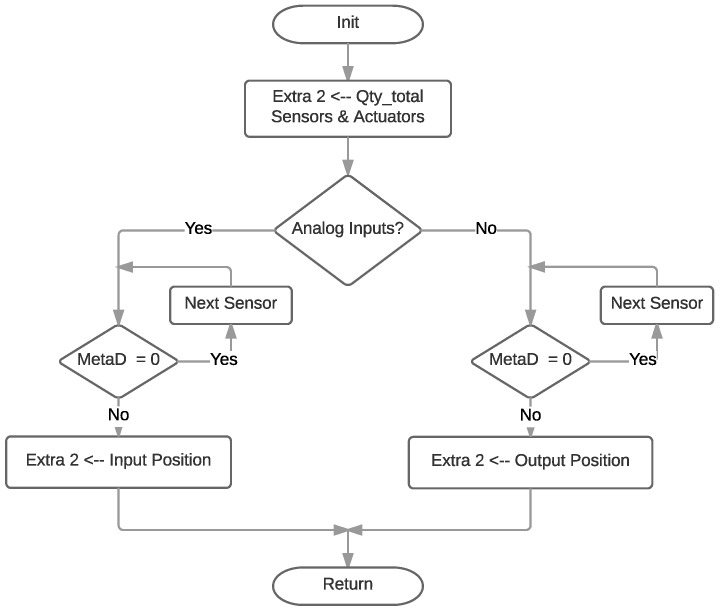
Extra 2 diagram flow.

The two bytes corresponding to Extra 2, which represent the Hardware Description/Data Value field, are filled according to the flow diagram shown in Figure 15. This field is mainly used for configuration. Only in the case of having to indicate a geolocation point, Extra 2 will join Extra 3 to provide a 32-bit data field. In Configuration Mode, the first byte of Extra 2 contains the total amount of transducers, while the second byte indicates the position of the transducers through a bitmap: if a bit is zero, it indicates that in that position there are no active transducers.

**Figure 16:**
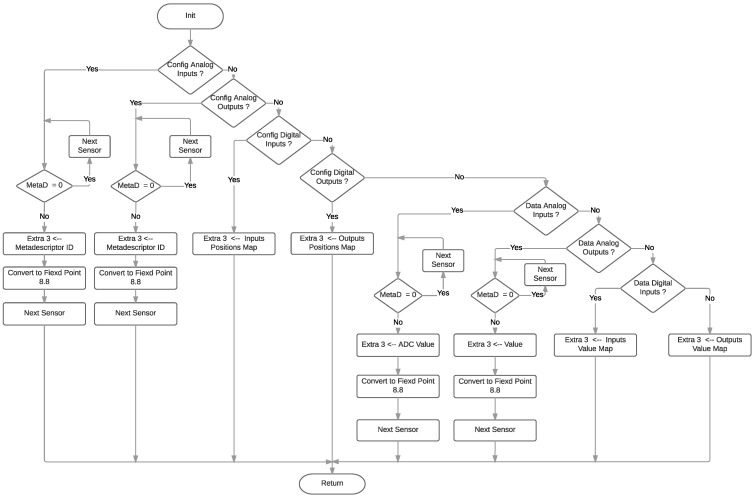
Extra 3 diagram flow.

Finally, Figure 16 shows the flow diagram followed to fill the Extra 3 byte, either with sensor values, meta-descriptors or, as it was previously mentioned, joining Extra 2 to provide a 32-bit field for geolocation coordinates. The digital information is provided through a bitmap, while the analog data make use of an 8.8 fixed-point format that is also used in standard Eddystone TLM frames.

## 5. Experiments

In this Section it is compared the performance of a beacon that makes use either of the LP4S-6 protocol or of a GATT server. For the sake of brevity, it was only evaluated LP4S-6, the most restrictive protocol of the LP4S family. The comparison was carried out in terms of latency and power consumption, which were evaluated in eight different scenarios: before the Android application performed a “characteristic connect” with the beacon, after carrying out such a connection, and for an Eddystone modified with the proposed protocol for beaconing intervals of 100 ms, 250 ms, 500 ms, 1000 ms, 2000 ms and 5000 ms. For all the intervals the physical scenario was identical, existing line-of-sight between the beacon and the smartphone, and a 3-m distance between both. The impact on the latency of such a transmission distance is negligible (the round-trip time is around 20 ns), and even for 100 m (BLE’s theoretical maximum distance), the round-trip time is less than 1 μs, while the obtained latencies measurements were in the order of seconds. Moreover, distance is not relevant for consumption measurements, since power is fixed (adaptive power transmission schemes are not used).

### 5.1. Experimental Setup

**Figure 17:**
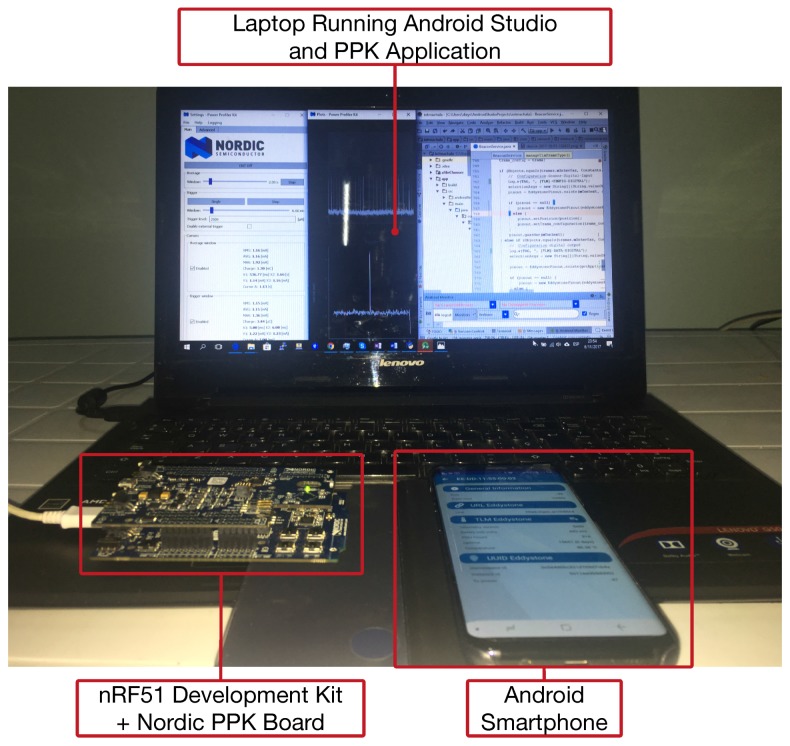
Experimental setup.

Figure 17 shows the elements involved in the experiments: a Nordic nRF51 development kit (nRF51-DK) that acts as BLE beacon, a Nordic Power Profiler Kit (PPK) that is plugged into the development kit for measuring current consumption, an Android smartphone and a laptop executing Android Studio and Nordic Power Profiler software kit. The main specifications of all these elements are described in Table 4.

### 5.2. Latency Measurements

Latency is key when collecting sensor data in certain applications and it is also a useful measure on the user experience. In the experiments presented in this article, latency is defined as the time that a system or a user has to wait to receive the value of a sensor after requesting it. To obtain the latencies, the Android Studio log was used, since it allows for calculating the time difference between the events related to when the user presses a button to get the current value of a parameter and when such a value is ready to be displayed on the smartphone screen. Android Studio log timestamps are expressed in milliseconds, which give enough precision for the designed experiments.

**Figure 18:**
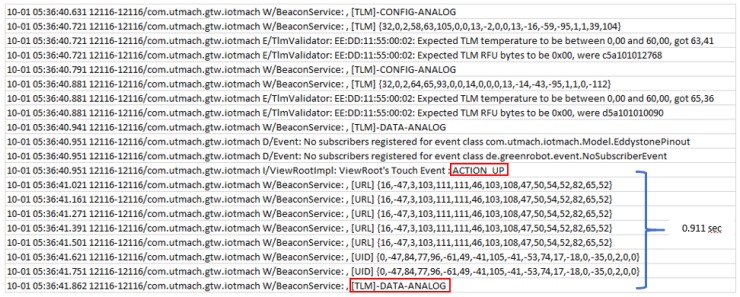
Example of Android log for measuring latency.

To carry out the measurements, a specific Android application was developed. The application sends certain text tags to easily identify events within the log. Thus, the measurement of the latency when establishing a connection is signaled with the tags “ACTION UP” (which is sent when pressing a button in the Android app) and “[CHR]-DATA-DIGITAL” (which indicates that data is ready to be shown on the screen). For the experiments with the LP4S-6 protocol, the same “ACTION UP” tag is first sent, but the second tag is “[TLM]-DATA-ANALOG”, which also indicates that there are sensor data ready to be displayed. Therefore, the difference between the timestamps associated with the arrival of both tags is the estimated latency. For instance, Figure 18 shows part of a log used to obtain the latency from a beacon. The log shown in such a Figure includes several tags related to Eddystone URL, UID and TLM frames. The tags marked with the red boxes are involved in the calculation of the latency that in the example was 0.911 s.

**Figure 19:**
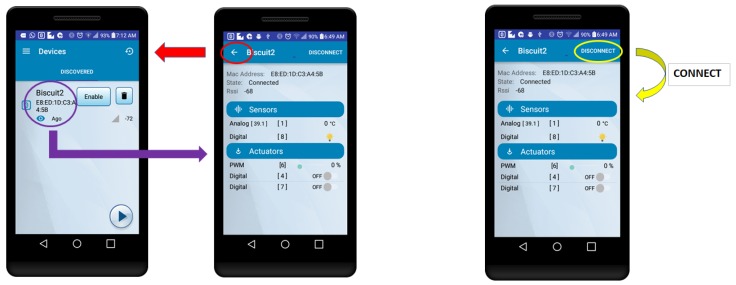
Establishing a connection by changing windows (**left**) or using the same window (**right**).

**Figure 20:**
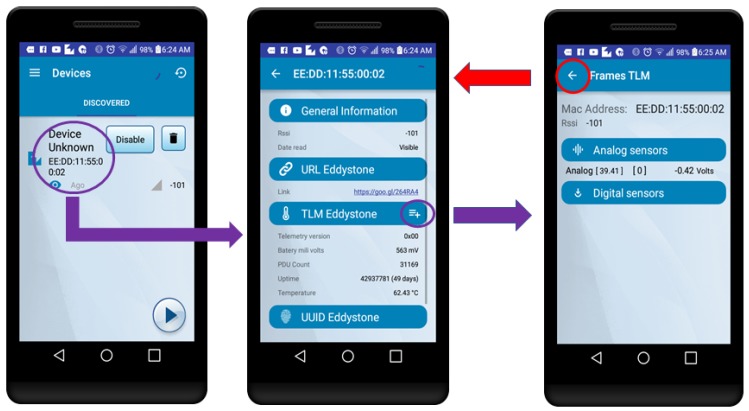
Accessing the embedded information through the Android app.

In addition to obtaining a detailed log of all the events, Android Studio allows for capturing real-time screenshots, such as the ones included in Figure 19 and Figure 20, which help to clarify the actions taken in every experiment. Figure 19 shows the actions performed when measuring the latency in the case of establishing a connection with the BLE mote. In such a Figure, in the use case on the left, the user selects one of the discovered BLE motes and a new screen is opened to interact with the mote, either to send commands or to read sensor values. Therefore, the latency would be the elapsed time since the user selects a mote in the first screen, until the sensor value is displayed on the second screen. In Table 5, this scenario is referred as “Connect changing windows”.

In Figure 19, on the right, it is illustrated a scenario where the sensor data are shown on the same screen after connecting. Thus, in this case, the latency would be measured since the user presses in the app the button to connect, until the value of the sensor is updated on the screen. In Table 5 the results associated with this scenario are referred as "Connect same window".

Regarding the latency measurements for the beacon-based experiments, they were performed through the same Android application, which is also able to disassemble TLM frames from an Eddsytone in order to obtain the sensor values inserted following the proposed protocol. Once the user selects the discovered beacon (in Figure 20, on the left), a new window is opened showing the standard Eddystone information of a TLM frame, but when the “+” button is pressed (marked with a circle) another window opens (in Figure 20, on the right) showing the sensor values. In this case, the latency is measured from the moment the user presses the “+” button, until the sensor values are shown on the screen. Note that such a way of measuring does not require to connect to the beacon, so the user has just to wait for the arrival of the proper TLM frame.

**Figure 21:**
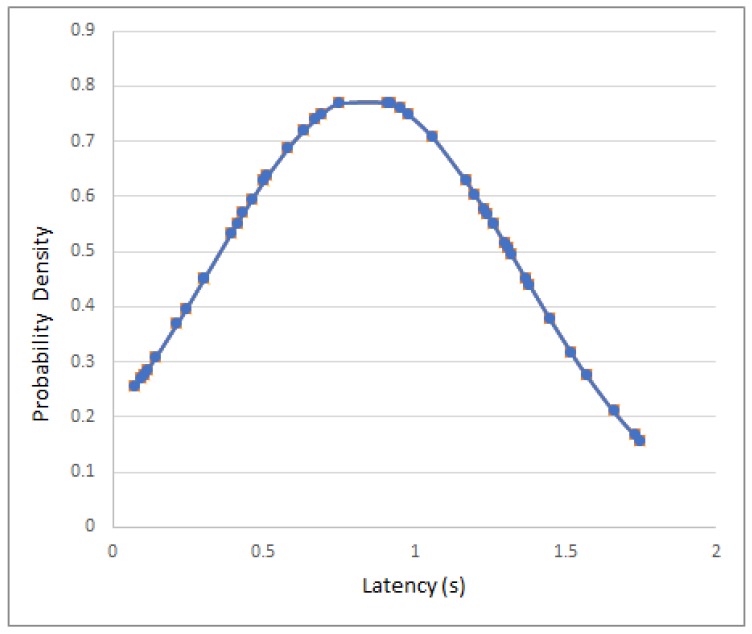
Latency distribution for a 100 ms beaconing interval.

For obtaining the latencies, up to 50 measurements were averaged for each scenario. It is interesting to point out that the obtained latencies followed a normal distribution. For example, in Figure 21 it can be observed the latencies obtained when evaluating a mote that used a 100 ms beaconing interval: the latencies can be modeled as a Gaussian bell curve with mean equal to 0.834 s and a standard deviation of 0.511 s. It is important to note that such curves can be used to model the behavior of the motes and then generate artificial samples from the fitted distributions to perform Monte Carlo simulations.

Table 5 shows the latencies obtained for the eight considered scenarios. In the case of the beacon tests, it can be observed that latency grows as the beaconing interval increases. The results allow for concluding that beaconing intervals of 100 ms and 250 ms are useful for real-time or quasi real-time applications, but larger intervals are not suitable for such applications. Regarding the GATT experiments (i.e., when a connection is established with the mote), it can be stated that the connection performed in the “changing windows” scenario is clearly faster than the “same window” alternative. This is due to the fact that the “same window” approach has to first discover and then connect to the mote, while in the “changing windows” version, the elapsed time is measured after a mote has already been discovered.

### 5.3. Mote Power Consumption

In order to obtain the power consumption of the mote in each of the eight previously proposed scenarios, the Nordic PPK was used. Such a kit consists of a dedicated board that is first inserted into the nRF51-DK and then to a computer through the USB. Nordic’s PPK desktop application is executed on the computer, which allows for obtaining accurate current consumption measurements of the mote. The PPK interface eases power consumption analyses by including tools to set time windows, to obtain the average consumption in an interval, or to zoom in and out. In addition, the captured data can be saved into a CSV file in order to perform a deeper analysis with other software tools like Matlab.

**Figure 22:**
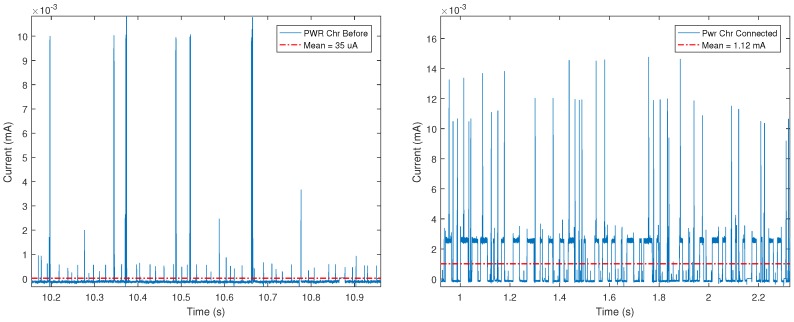
Power consumption for GATT before connecting (**left**) and GATT when connected (**right**).

**Figure 23:**
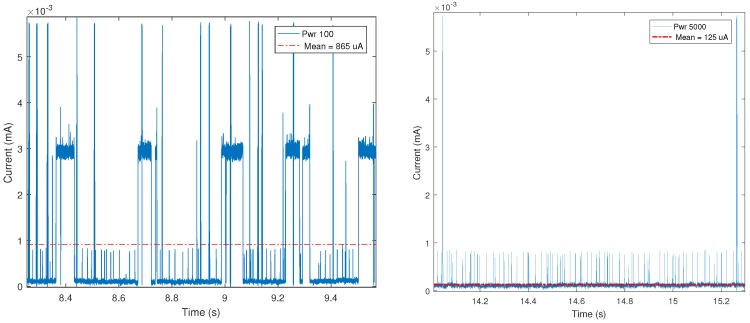
Power consumption for beacon intervals of 100 ms (**left**) and 5000 ms (**right**).

Figure 22 and Figure 23 show significant examples of the current measurements obtained. In such Figures it can be observed a remarkable difference among the four cases shown. Figure 22 compares the consumption of a BLE mote based on GATT characteristics before and after connecting: it only consumes 35 μA when it is not connected and up to 15 mA when the connection is established, although the average consumption is actually 1.12 mA.

Figure 23 shows the consumption for a mote that uses beaconing intervals of 100 ms and 5000 ms. For a 100 ms interval, there are consumption peaks of up to 7.5 mA (the average is 865 μA), while with 5000 ms interval the measured peaks are similar (of up to 7 mA), but the average consumption descends to 125 μA.

Table 6 contains the current consumption obtained for different beaconing intervals and for the proposed GATT experiments. The second column shows the average current consumption, while the rest of the columns contain estimations on how many months a device would last with different kinds of commercial batteries. It should be clarified that this computation assumes that the batteries get completely discharged, which is not usually the case, since most batteries can only maintain a proper voltage until their capacity is between 20% or 30% of the total. Therefore, the expression used in Table 6 for calculating the battery life is:

(1) $$Battery\_life\;\left(months\right)=\frac{Battery\_capacity\;(mAh)}{\frac{Mote\_average\_consumed\_power\;(\mu A)}{1000}\times 24\times 30}$$

With respect to the measurements on the beacons, it can be easily observed how increasing the beaconing interval decreases significantly current consumption. In the best case, for a 5000 ms beaconing interval, a VNR1582 battery would last 55 months (roughly four and half years), which is approximately four times more than the 12 months that a GATT-based mote would achieve on average with the same battery.

In the case of the GATT experiments, the stand-by current is so low that even with the smallest battery the device would last several months in such a mode. Energy consumption is clearly higher when the mote is connected, due to the power consumed by the BLE transceiver, but note that, in general, motes remain most of the time in stand-by waiting for connections.

### 5.4. Analysis of the Results

The previous subsection showed relevant conclusions regarding the latency and the current consumption, but it is interesting to dig deeper for a better understanding of the relationship between both factors.

**Figure 24:**
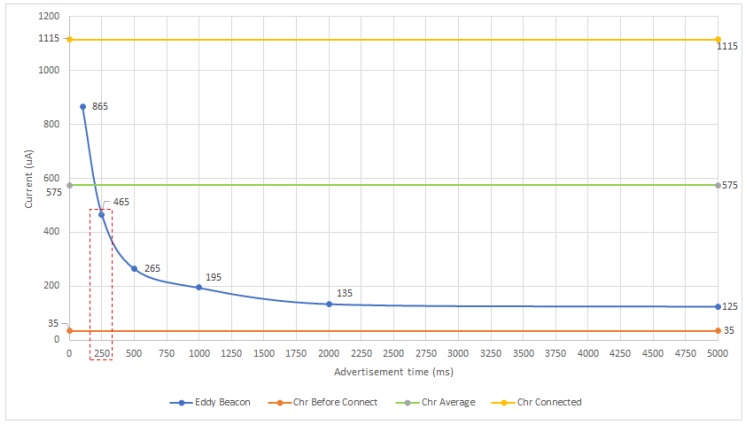
Current consumption versus beaconing interval for the performed experiments.

First, it is helpful to visualize the results from Table 5 and Table 6 in the way that they are depicted in Figure 24. In such a Figure the straight lines represent the current consumption for the different GATT experiments, being the one at the bottom the one related to the consumption when the mote is disconnected, and the one at the top, the one that represents the current consumed when a connection is established. Thus, this latter straight line would represent the case in which the mote is always connected, which in practice is not efficient, but that could be the best scenario when several users access sequentially to the same device. In contrast, the central line is the average of the two other straight lines and represents a more realistic situation where one or more users connect sporadically to a mote.

Regarding the ‘Eddy Beacon’ curve, it shows the current consumption behavior of a beacon for the different advertising intervals tested. Comparing the curve with the three reference levels from the GATT experiments, it can be concluded that the beaconing solution that implements LP4S-6 obtains better results than when using GATT characteristics in real scenarios with one or several users connecting sporadically. In fact, all the consumption values obtained except for the one associated to a 100 ms beaconing interval, are below the central line (‘Chr Average’). The gains are even larger when it is possible to increase the beaconing interval, although it seems that, with the tested hardware, there is a consumption floor after 2000 ms after which the current drawn does not decrease significantly.

It is also important to realize that the protocols of the LP4S family do not limit the number of concurrent users and the consumption of the mote is not influenced by such users, since a connection to the device is not required to obtain the sensor values and, therefore, all the users receive in parallel the data broadcast. This fact means that, when multiple users access a mote requiring a connection, the current would be close to the straight line (‘Chr Before Connect’) shown in Figure 24 (1115 μA), what implies that even the 100 ms beaconing interval alternative (865 μA) would be more efficient in terms of energy.

**Figure 25:**
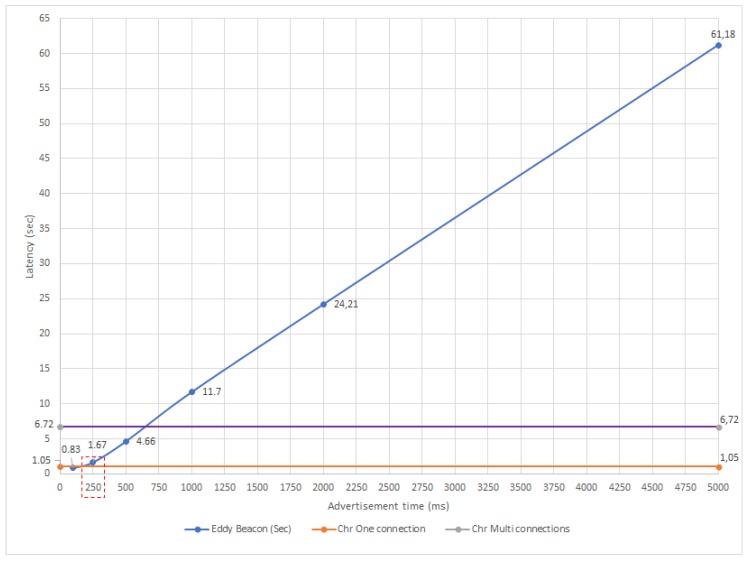
Latency versus advertisement time.

With respect to the latency, Figure 25 allows for comparing the results for GATT-based and beacon-based motes in relation to the beaconing interval. Again, the straight lines serve as reference and represent the latencies shown in Table 5 for the GATT experiments, while the ‘Eddy Beacon (Sec)’ curve contains the data for the LP4S-6-based proposed approach. Thus, the ‘Chr One connection’ line represents the best scenario achieved when only one user is connected to the mote, while the ‘Chr Multiconnections’ line represents the latency when several users connect to the same mote. In Figure 25 it can be also observed that the LP4S-6 beacon shows for 100 ms and 250 ms a latency close the best GATT scenario, being even smaller in the 100 ms case. Nonetheless, for 1000 ms, 2000 ms and 5000 ms beaconing intervals, it is clearly exceed the 6.72 s of latency obtained for the multi-user GATT scenario.

Finally, note that in Figure 24 and Figure 25 there is a dotted rectangle that indicates the solution that gives the best trade-off between latency and energy consumption. However, it is important to emphasize that the selection of the mote communications protocol is highly dependent on the application. For instance, in a telemetry application with no power restrictions, where only one concurrent user is connected to the mote and that requires low sampling rates, both the GATT approximation and the 100 ms and 250 ms beaconing approaches would be recommended. However, in the same situation, if there are multiple users, GATT would not be a good choice. Nonetheless, in general, for remote control and telemetry applications, the use of GATT for establishing a connection is usually the best option.

A different scenario arises when motes are powered with batteries and installed in remote locations where regular maintenance is difficult or expensive. In such a scenario, beacon-based approaches tend to be the best alternative. Moreover, the parameters to be sensed determine the beaconing interval and the battery can be chosen following the results obtained in Table 5 and Table 6, and taking the mote dimensions and maximum weight into account. An example of the described scenario can happen in a precision agriculture application, where a 5000 ms interval would be enough to measure factors like ambient temperature or soil moist, which in most situations change slowly. In addition, in such scenarios, usually there are not size restrictions, so a large battery could be embedded into the mote.

## 6. Conclusions

This paper presented the LP4S family of lightweight protocols, which are focused on providing low-latency and low-power consumption communications. Furthermore, LP4S enables mote self-detection and self-registration through plug-and-play mechanisms for IoT telemetry systems. The use of BLE motes for telemetry involves two types of connectivity: they can be either connected directly to a GATT server via characteristics or they can be used as beacons with the help of the LP4S protocol. To determine which type of connectivity is the best to be used in different contexts and applications, several experiments were carried out. It was demonstrated the feasibility of using the LP4S-6 protocol, which was embedded into a commercial Eddystone beacon. The results showed the superiority of the proposed solution in terms of latency and energy consumption when multiple users connected to a mote. Moreover, the findings also suggested the use of the LP4S-6 protocol for scenarios where latency was not a restriction, but low-energy consumption was essential. Finally, it is worth mentioning that, although the developed protocol family was tested within a protocol as restrictive as the one defined by Eddystone beacons, it can also be implemented for other similar IoT devices, providing efficient and fast communications.

## Figures and Tables

**Table 1:** Related works on the different layers of the IoT Stack.

Layers	Protocols	References
Application	IoT, WoT, SWoT, Device Management, Analytics, Business Process	[49,52,53]
Presentation	Binary, LWM2M, XML, JSON, IPSO Objects, SOAP	[49,50,52,53]
Session	CoAP, MQTT, XMPP, AMQP, HTTP, Websocket	[28,33,47,48,49]
Transport	UDP, TCP, IPSP, GATT, GAP, DTLS, TLS	[32,44,45,46]
Network	IPV6/IPV4, RPL, IPSP, 6LoWPAN Adaptation	[39,40]
Physical/Link	IEEE 802.15.4, IEEE 802.3, Ethernet, Wi-Fi, BLE, Z-Wave, DASH7, RFID	[34,35]

**Table 2:** Comparison of relevant commercial beacons.

Beacon	Chipset Manufacturer	Protocols	Battery	Battery Life (Months)	Cost (US $)
Estimote [68]	Nordic	iBeacon or Eddystone	CR2450	21.4	3 for $59.00
BKON [69]	Nordic	iBeacon or Eddystone	2 × AAA	28.9	$30.00
RadBeacon Dot [70]	Nordic	iBeacon, AltBeacon or Eddystone	CR2032	5.00	$14.00
Konkat [71]	Nordic	iBeacon or Eddystone	CR2477	24.3	3 for $60.00
OpenBeacon BL [72]	Nordic	Eddystone	CR2032	N/A	$25.00
BlueSense [73]	Bluegiga	iBeacon	CR2450	12.7	£19.99
BlueCats [74]	Bluegiga	iBeacon or Eddystone	2 × AA	23.1	3 for $89.00
RedBear Beacon B1 [75]	TI	iBeacon	2 × AAA	21.00	$21.00
SWIRL [76]	TI	iBeacon or Eddystone	4 × AA	72.00	N/A
Glimworm [77]	TI	iBeacon	CR2450	11.0	4 for 99.00
Gimbal [78]	Qualcomm	iBeacon or Eddystone	4 × AA	16.4	$5.00

**Table 3:** Main tags used in the JSONs.

Tag	Description	Value (Example)
MAC	Device MAC	“00:11:22:33:44:55”
Name	Device name	"Z1-T1"
Manufacturer	Manufacturer Identification code	“070000000001”
Location	Longitude, Latitude and Altitude	“(00, −00, 00)”
Description	Manufacturer, supplier, technology, others	“nRF51-Dk”
Qty_all	Total transducers (sensors + actuators)	“8”
Metad	Transducer Meta descriptor	“1.0”
Interface	Interface + signal + position	“ina7”
Button	Push button event	“1”
Pos_dig_in	Position map (Digital inputs)	“3”
Pos_dig_out	Position map (Digital outputs)	“128”
Pos_ana_in	Position map (Analog inputs)	"5"
Pos_pwm_out	Position map (PWM outputs)	“136”
Package_id	Package write reference	“1005”
Value	Data value (Read or Write)	“23.5”

**Table 4:** Main specifications of the experimental setup elements.

Experimental Setup Element	Main Specifications
Laptop	Lenovo 80E502A5SP G50-80: 15.6", 16 GB RAM, 1 TB HDD
	Intel^®^ Core™i7-5500U CPU @ 2.40 GHz
	OS version: Windows 10-Pro x64 bits
Android Studio 2.3.3	AI-162.4069837, built on 6 June 2017
	JRE: 1.8.0.112-release-b06 amd64
	JVM: OpenJDK 64-Bit Server VM by JetBrains s.r.o
Nordic nRF51 development kit (nRF51-DK)	SoC: nRF51822, 2.4 GHz multi-protocol device, 32-bit ARM^®^
	Cortex™M0 CPU with 256 kB/128 kB flash + 32 kB/16 kB RAM
Nordic PPK (Power Profiler Kit)	Board: PCA63511
	Software: nRF6707-SW v1.1.0
	Python version: 2.7.13, Pyside: 1.2.4, Pyqtgraph: 0.10.0, Numpy: 1.13.1, Pynrfjprog: 9.6.0
Smartphone	Samsung Galaxy S-8, 4 GB RAM, 64 GB (UFS 2.1) ROM
	Model: SM-G950F
	Android version: 7.0 (Nougat)
	Processor: Exynos 8895, 2.3 GHz Quad + 1.7 GHz Quad, 8 Cores (Octa-Core)

**Table 5:** Latency results for the different proposed experiments.

Experiment		Latency (s)
Beaconing Interval	100 ms	0.83
	250 ms	1.67
	500 ms	4.66
	1000 ms	11.7
	2000 ms	24.21
	5000 ms	61.18
GATT	Connect Changing Windows	1.05
	Connect Same Window	6.72

**Table 6:** Current consumption results.

Experiment		Power (μA)	CR2032 240 mAh (Months)	CR2477 1000 mAh (Months)	VNR1582 5000 mAh (Months)
Beaconing Interval	100 ms	865	0.385	1.606	8.028
	250 ms	465	0.717	2.987	14.934
	500 ms	265	1.258	5.241	26.205
	1000 ms	195	1.709	7.123	35.613
	2000 ms	135	2.469	10.288	51.44
	5000 ms	125	2.667	11.111	55.556
GATT	Connect Changing Windows	1115	0.299	1.246	6.228
	Connect Same Window	1115	0.299	1.246	6.228
	Before Connecting	35	9.524	39.683	198.413
	Average	575	0.58	2.415	12.077

## Abbreviations

The following abbreviations are used in this manuscript:

AMQP	Advanced Message Queuing Protocol
BLE	Bluetooth Low Energy
CoAP	Constrained Application Protocol
DTLS	Datagram Transport Layer Security
GATT	Generic Attribute Profile
HF	High Frequency
IoT	Internet of Things
ISR	Interrupt Service Routine
FPGA	Field-Programmable Gate Array
LF	Low Frequency
LP4S	Lightweight Protocol for Sensors
LWM2M	Lightweight Machine-to-Machine
MAC	Medium Access Control
MQTT	Message Queue Telemetry Transport
PPK	Power Profiler Kit
PWM	Pulse-Width Modulation
QoS	Quality of Service
REST	Representational State Transfer
SBC	Single-Board Computer
SoC	System-on-a-Chip
SSL	Secure Sockets Layer
TLM	Telemetry
UHF	Ultra-High Frequency
WoT	Web of Things
WSN	Wireless Sensor Networks
WTM	Web Thing Model
XMPP	Extensible Messaging and Presence Protocol
